# Faculty’s Perception of Their Role as a Medical Teacher at Qassim University, Saudi Arabia

**DOI:** 10.7759/cureus.9095

**Published:** 2020-07-09

**Authors:** Shazia Nawabi, Safia Shoeb Shaikh, Muhammad Qasim Javed, Arham Riaz

**Affiliations:** 1 Medical Education, Qassim University, Buraidha, SAU; 2 Radiology, Qassim University, Buraidha, SAU; 3 Conservative Dentistry and Endodontics, Qassim University College of Dentistry, Buraidha, SAU; 4 Public Health Dentistry, Academy of Continuing Health Education and Research (ACHER), Islamabad, PAK

**Keywords:** professional roles of medical teacher, dental faculty, perceptions

## Abstract

Purpose

Medical teaching is a highly demanding and complex task. The advanced integrated curriculum and modern educational practices demand the roles of the medical teacher be redefined. This study was designed to understand the perceptions of the faculty of the Dental College, Qassim University, about their key roles as a teacher. These perceptions can be used to design faculty development workshops to enhance the awareness of the faculty about their educational responsibilities and achieve their potential.

Methods

It was a cross-sectional descriptive survey conducted on the faculty of the College of Dentistry, Qassim University, KSA. The study used a validated 12-item e-questionnaire to measure the perceptions of faculty about their teaching roles.

Results

A total of 44 faculty members submitted the e-questionnaire. Most faculty members perceived the most important role of the medical teacher as an information provider (90%) in clinical settings, followed by an on-job role model (89%). The least important role perceived was curriculum evaluator (82%) followed by curriculum planner (79%).

Conclusion

The role of a medical teacher has extended beyond the boundaries of information providers. The faculty of Qassim University exhibited their awareness about modern-day medical education and recognized the most important role of a medical teacher to be not only an information provider but also an on-job role model and academic advisor to students.

## Introduction

The medical profession has become a stressful and highly demanding field due to significant changes that have taken place in response to advancements in medicine, the changing healthcare delivery system, and patients' needs and expectations [1-2]. This revolution in medical practice needs to be mirrored by similar changes in the education system and the acceptance of new roles by medical teachers in the context of developments in educational thinking.

Teachers are the backbone of an education system. They play a dynamic role in the learning of students and have a significant influence on the delivery of healthcare through the students they teach once they are in medical practice. A medical teacher’s commitment to teaching may vary from an occasional lecture to the regular supervision of clinical sessions, to responsibility as course director or curriculum planner, and to a full-time education position with a range of educational duties [3].

Therefore, it is critical for the medical teachers to be aware of the key roles they need to play and develop the skills, attitudes, and practices of a competent teacher in facilitating student's learning. This metacognition gives them a better understanding of their cognitive processes [4]. This, coupled with the personal satisfaction of being an effective teacher, is the motivation to become a better medical educator [5]. It is important to remember that the actual details of any curriculum matter little as compared to the selection of teachers. If the teachers are good, any system will work effectively, and if they are indifferent, even the best curriculum will fail to produce the desired outcomes [3]. Hence, the improvement in medical education is largely dependent upon the attitude of medical teachers toward their instructional practice and the learning of their students [5].

The teaching methodologies and how the learners are expected to acquire knowledge have been transformed significantly under the influence of educational theories and adult learning principles [6]. The aforementioned change has resulted in the emergence of novel educational terminologies; one such terminology utilized in curriculum design is ‘learner-centered education.’ With this paradigm shift, many medical colleges have shifted from a traditional ‘teacher-centered’ program to a ‘learner-centered’ curriculum [7-8]. Integrated teaching, community-based learning, problem-based learning, core curricula with electives, and more methodical planning of curriculum have been advocated. Vital trends of medical education in the ‘classroom,’ as well as ‘the bedside,’ are changing greatly with the incorporation of new educational approaches [9].

Historically, the major role of a teacher has been as ‘the provider of information.’ In recent years, the emphasis has been placed on students taking more responsibility for their own learning. Teachers are no more the sole provider of truth, but they should use their expertise to validate available information and guide students to find key issues that are relevant to their future practice. Therefore, currently, the teachers’ role is as ‘the facilitator of learning’ [10].

Additionally, in the present educational environment, teachers are expected to perform multiple roles that include managerial responsibilities comprising curriculum and course planning, implementation of the plan, and monitoring the process of education. Furthermore, the leadership style of the teacher is of great significance in developing and executing the teaching-learning program. Also, contemporary academic and professional literature has promoted the idea of reflection in teaching. Reflective practice helps in promoting professional growth and uncovering the hidden assumptions, beliefs, and values about teaching [11]. Likewise, positive role-modeling can enhance the students learning from the practice of communication skills and ethical issues by observing teachers. Subsequently, it helps in the inculcation of personal and professional growth, with the required ethical standards, among the medical students [12].

Medical teachers may perceive their roles differently, depending on several factors, such as their own educational background, the educational environment of the institute, and the level of their training as a medical teacher [13]. Several studies have been conducted globally to understand the perceptions of teachers about their roles and responsibilities [14-15]. These studies indicate that teachers perceive their roles differently in different institutes [16-17].

In the last two decades, the Kingdom of Saudi Arabia has also experienced a shift from the ‘teacher-centered curriculum’ to ‘the learner-centered curriculum’ by the implementation of curricular reforms, faculty development, and improving the learning environment [18]. There are thousands of teachers providing education in Saudi dental colleges. To the best of our knowledge, one study has been conducted at Qassim University, evaluating the perception of Saudi medical students about the qualities of effective teachers [19], but teachers’ perceptions about their own roles have not been studied yet in any dental college in Saudi Arabia.

The aim of this study was to understand the perceptions of faculty teaching at the Qassim University College of Dentistry about their roles as teachers. These perceptions can subsequently help in developing effective teaching and training strategies, allowing teachers to excel in their roles.

## Materials and methods

This is a cross-sectional, descriptive, e-questionnaire-based study conducted in Qassim University College of Dentistry from January to March 2019. The study used a 12-item validated e-questionnaire based on the identification of the 12 roles of a medical teacher. These roles were further grouped into six dimensions as presented in the model [1]. Baseline data included the name of the person (optional), gender, and designation in college. Participants were asked to mark the relative importance of each of the 12 roles on a four-point Likert scale, consisting of categories: (1) none (2) no idea (3) agree (4) strongly agree. Before distribution, the e-questionnaire was pilot tested on five teachers from Qassim Dental College, not included in the study. The major purpose of pilot testing was to see the clarity of language and the understanding of the terms used in the e-questionnaire.

Ethical approval was obtained from the ethics board of Qassim University (ST/49/2018). All participants were informed about the content and intent of the study and were assured about the maintenance of confidentiality. The data were collected through Google forms sent to the official e-mails of all faculty members from January to March 2019. Demonstrators were not included in the study, as they have relatively less experience in teaching. The total sample consisted of 85 faculty members using convenience sampling. Out of 85 faculty members, only 44 responded by filling the e-questionnaire after two rounds of follow-up reminders. Data analysis was carried out using the Statistical Package for Social Sciences (SPSS) software package version 24 (IBM Corp., Armonk, NY).

## Results

There were 44 respondents with an overall e-response rate of 50.8%, and the male to female ratio of respondents was 1.3:1 (Table 1).

**Table 1:** Demographic details of the participants

Demographic Details of the Participants				
Mean age			41 years	
Gender				
Male	26			
Female	18			
Designation				
Professors	Associate professors	Assistant professors		Lecturers
4	9	18		13

**Figure 1:**
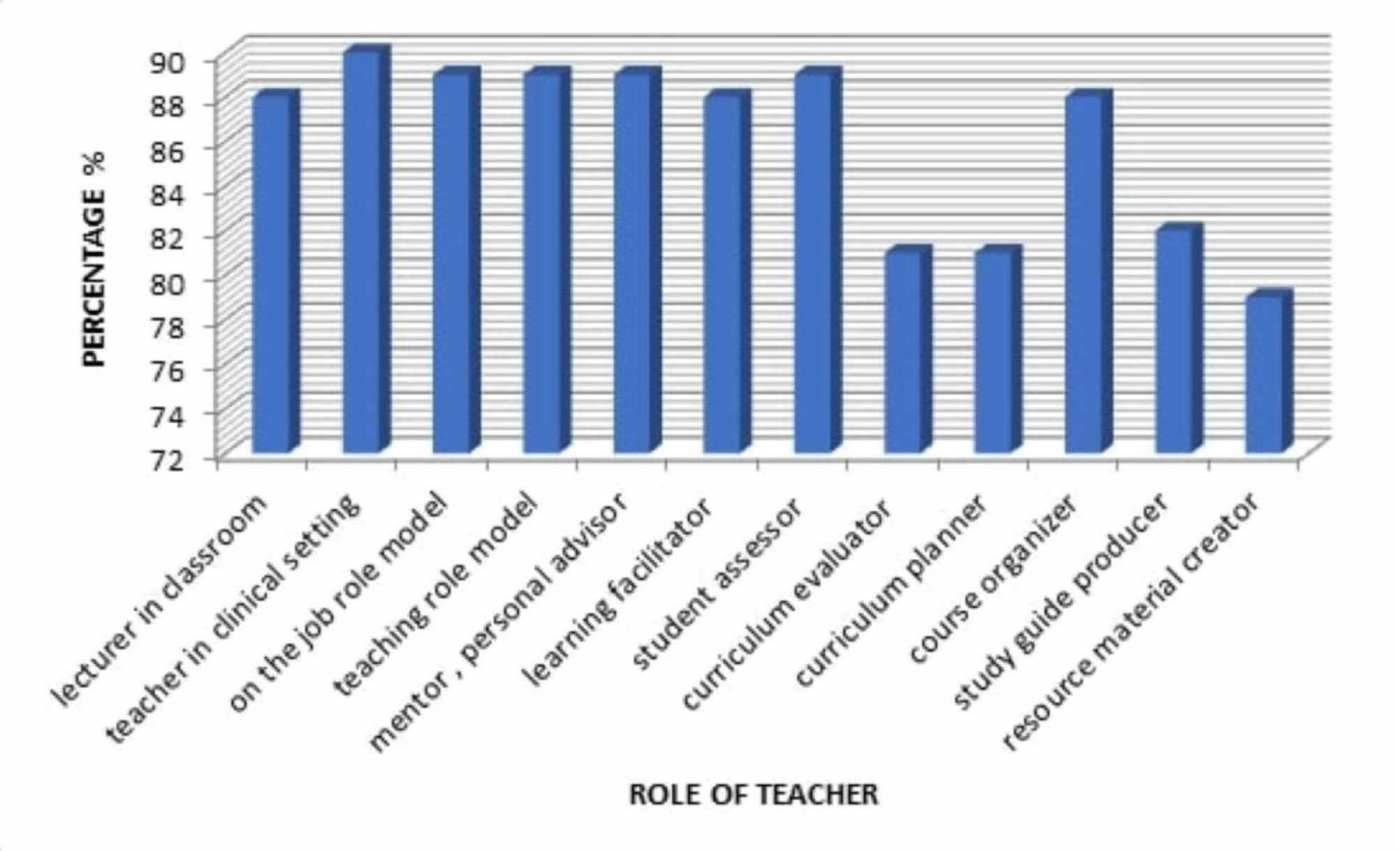
Comparative distribution of responses according to the degree of importance of each role

As far as individual roles were concerned, the role perceived as the most important was information provider in clinical settings (90%) while the least important roles perceived were study-guide provider (82%) curriculum evaluator and planner (81%) and resource developer (79%) (Figure 1).

**Figure 2:**
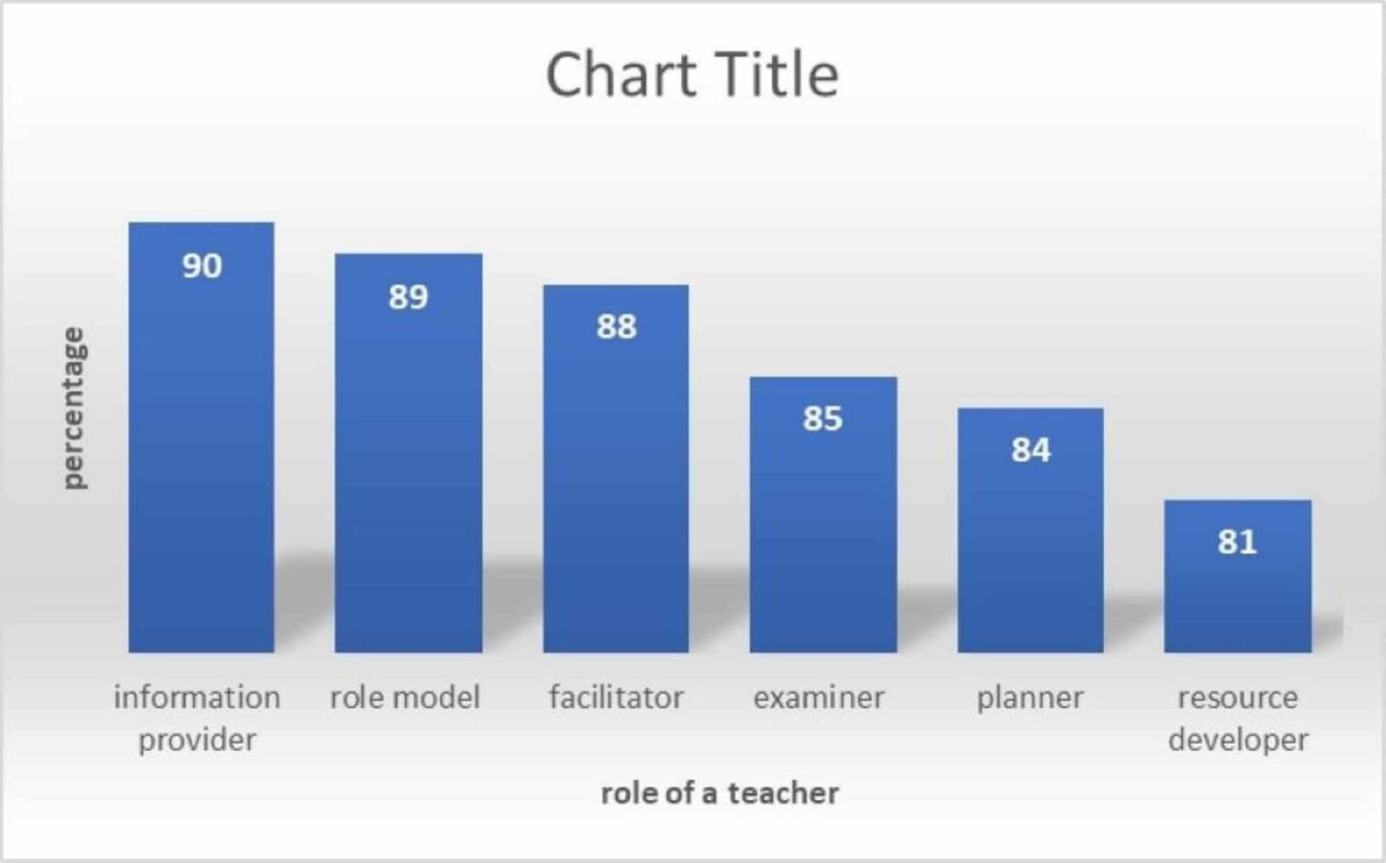
Relative importance of each dimension as perceived by faculty

Next, the individual roles were grouped into the six dimensions of medical teaching, and Figure 2 presents the relative importance of each dimension in percentage value. Whereas Table 2 provides the relative importance of each role individually as well as in dimensions. The results of the current study highlighted the fact that the majority of medical teachers perceived the traditional role of a medical teacher as a basic source of information provider as most important. Generally, roles of medical expertise were given more weightage as compared to roles with educational expertise.

**Table 2:** Relative importance of each role individually and in groups as dimensions

Roles of teacher	Percentage value	Dimensions	Percentage value
Classroom lecturer	88 %	Information provider	89%
Teacher in the clinical setting	90 %		
Role model as a clinician	89 %	Role model	89%
Role model in teaching	89 %		
Mentor/academic advisor	89 %	facilitator	88.5%
Learning facilitator in small groups	88 %		
Formal examiner of students	89 %	Assessor	85%
Curriculum evaluator	81 %		
Curriculum planner	81 %	Planner	84.5%
Course organizer	88 %		
Production of study guides	82 %	Resource developer	80.5%
Developing learning resources	79 %		

Data were further analyzed by the gender and designation of the faculty. There were only minor differences in the perceptions of male and female faculty members, as both perceived the most vital role of a medical teacher as an information provider in the clinical setting and the least important role as study guide provider. The designation of the faculty was taken as an indicator of teaching experience. Faculty at the ranks of full professor and associate professor were combined as having more experience than assistant professors and lecturers. Only minimal differences were observed in the perceptions of the two groups about the roles of the teachers.

## Discussion

Teaching in the medical profession requires proﬁcient knowledge of motivating the learner and assessing their competence. In addition, the skills to deal with the competing demands of patient care, research, and education are equally important [20]. In fact, a teacher is not only a role model who has an influence on every facet of students' growth and on developing their innate potential but is also a motivator, guide, and friend [21]. The responsibility of a medical educator is to convey and decode the language of medical science into the minds of learners. Besides, the medical teacher of today is also responsible to enable and empower the learner to emerge as a competent physician who is ready to take on the challenges of the rapidly changing world as a health leader [22].

Association for Medical Education in Europe (AMEE) Guide no 20 describes the responsibilities of the teacher as 12 roles arranged in six dimensions [1]. The model has been widely used by teachers to assess their personal roles, by institutions to plan educational activities, by faculty developers in education courses, and researchers in the field of medical education [23].

The present study has used the same model to assess the perceptions of the multinational faculty of a Saudi university. In our study, the most important role perceived was that of an information provider in a clinical setting, followed by an on-job role model. This is in accordance with the results of a study done in Thailand where three highly-rated roles were clinical teacher, on-job role model, and lecturer [24]. Likewise, Nawabi et al., in their study, noted that the faculty of Pakistani medical colleges perceived the most essential role of the medical teacher as an information provider [13]. They further compared their findings with that of Dundee University and found statistically significant differences between the perceptions of both faculties. Differences were attributed to an educational environment, the training of faculty, and the influence of national cultures and material wealth [13]. Two recent studies also concluded that the maximum number of faculty members perceived the most important role of a medical teacher as a provider of information in a clinical setting and the least important as planner and evaluator [25-26]. The results of all these studies reflect the fact that in an Asian society, the teacher is still highly valued as an information provider and a role model, and other roles, especially teachers as a resource provider and curriculum planner and evaluator are given less preference. Studies further concluded that it is most likely due to cultural reasons or a carry-over effect of the basic education system of Asian societies. Many developing countries are still using traditional curricula due to a lack of infrastructure and resources required to plan and implement modern integrated, student-centered curriculums.

Our study further indicates that roles with medical expertise are given more priority than with educational expertise. These results contrast with studies done by Reuler and Nardone, which emphasized the managerial role, consisting of planning and controlling the curriculum, and leadership style as the most important roles of a teacher in the current educational context [21]. Another study highlighted the importance of the following six roles of medical teacher; facilitator, information provider, curriculum developer, assessor and assessment creator, role model, and resource developer. Other roles defined included professional expert, faculty developer, researcher, and administrator [27].

The results of the majority of these studies done around the world demonstrate that the nature of the role of medical teacher may vary from specialty to specialty and from one learning environment to another, but the basic role of the teachers has always been vital as a provider of information and a role model. Nevertheless, in the current study, the faculty of Qassim University College of Dentistry perceived other roles of a medical teacher to be almost equally important except the role of a medical teacher as a resource developer. This level of awareness may be attributed to regular faculty development workshops organized by dental education and quality units to help in capacity building. When results were compared by gender, there was no statistically significant difference in the perceptions of the two groups. Senior faculty members (professors and associate professors) gave more importance to roles with educational expertise, especially a teacher as an assessor and curriculum evaluator, whereas relatively less experienced teachers (assistant professors and lecturers) were more focused on traditional roles with medical expertise.

The limitation of the present study is that it was conducted in one public sector dental college, which does not mean it represents the perceptions of all Saudi medical teachers. Faculty perceptions about their roles may vary in different educational environments; therefore, it is important to conduct a larger national-level multicentre study to understand faculty perceptions in a variety of setups. A range of qualitative studies may also be needed to better understand the in-depth reason for the teacher's perception of their roles. Once perceptions of medical teachers about their roles are identified, medical institutes can prepare faculty development programs to provide training to all faculty members.

## Conclusions

A teacher is pivotal to the success of any educational program. The role of a teacher in the educational process is changing. Teachers must reflect upon their performance so that average teachers can transform into good teachers, good teachers into the best, and the best teachers can become inspiring. The present study has concluded that the faculty of Qassim University exhibited their awareness of and familiarity with modern-day medical education.
